# Nanodiamond as a Cytokine Sponge in Infectious Diseases

**DOI:** 10.3389/fbioe.2022.862495

**Published:** 2022-04-04

**Authors:** Wonbeak Yoo, Wonhwa Lee, Hong Nam Kim, Jiyoung Jeong, Hee Ho Park, June Hong Ahn, Dana Jung, Juheon Lee, Ji-su Kim, Seung Whan Lee, Wan-Seob Cho, Seokho Kim

**Affiliations:** ^1^ Environmental Disease Research Center, Korea Research Institute of Bioscience and Biotechnology, Daejeon, South Korea; ^2^ Department of Chemistry, Sungkyunkwan University, Suwon, South Korea; ^3^ Brain Science Institute, Korea Institute of Science and Technology (KIST), Seoul, South Korea; Division of Bio-Medical Science and Technology, KIST School, Korea University of Science and Technology, Seoul, South Korea; ^4^ Department of Health Sciences, The Graduate School of Dong-A University, Busan, South Korea; ^5^ Department of Bioengineering, Hanyang University, Seoul, South Korea; ^6^ Division of Pulmonology and Allergy, Department of Internal Medicine, College of Medicine, Yeungnam University and Regional Center for Respiratory Diseases, Yeungnam University Medical Center, Daegu, South Korea; ^7^ Primate Resources Center, Korea Research Institute of Bioscience and Biotechnology, Jeongeup, South Korea; ^8^ Institute of Plasma Technology Research, Korea Institute of Fusion Energy, Gunsan-si, South Korea; ^9^ Department of Medicinal Biotechnology, College of Health Sciences, Dong-A University, Busan, South Korea

**Keywords:** nanodiamond, cytokine release syndrome, adsorption, infectious diseases, multiple-organ failure

## Abstract

Cytokine release syndrome (CRS) is a systemic inflammatory response resulting in overexpression of cytokines in serum and tissues, which leads to multiple-organ failure. Due to rapid aggravation of symptoms, timely intervention is paramount; however, current therapies are limited in their capacity to address CRS. Here, we find that the intravenous injection of highly purified detonation-synthesized nanodiamonds (DND) can act as a therapeutic agent for treating CRS by adsorbing inflammatory cytokines. Highly purified DNDs successfully inactivated various key cytokines in plasma from CRS patients with pneumonia, septic shock, and coronavirus disease 2019 pandemic (COVID-19). The intravenous injection of the DND samples in a mouse sepsis model by cecal ligation and puncture significantly improved survival rates and prevented tissue damage by reducing the circulating inflammatory cytokines. The results of this study suggest that the clinical application of highly purified DND can provide survival benefits for CRS patients by adsorbing inflammatory cytokines.

## Introduction

In some cases, the immune system of people infected with pathogens is overstimulated, which results in the overproduction of inflammatory cytokines and chemokines by immune cells such as neutrophils and monocytes (Van Linthout et al., 2014). Then, a vicious cycle of more inflammatory mediators and more recruited inflammatory cells can aggravate inflammation at the site of infection as well as systemic circulation (Schroder et al., 2006). In a worst-case scenario, it can cause a systemic inflammatory overreaction known as fatal cytokine release syndrome (CRS), which results in septic shock and multiple organ failure (Chousterman et al., 2017; Shimabukuro-Vornhagen et al., 2018). The recent coronavirus disease 2019 pandemic (COVID-19) presents an example of a disease that comes with a high risk of CRS (Ma et al., 2020; Yang et al., 2020). Therefore, timely intervention to reducing systemic circulating inflammatory cytokines is critical for managing CRS.

There are several strategies for CRS management including blood filtration, treatment with corticosteroids, and the use of antagonists for inflammatory cytokines. Blood filtration therapy is one of the most widely used clinical practices (Ma et al., 2020). However, a complicated facility for blood filtration is required, although it is common and relatively straightforward. The use of corticosteroids, a class of steroid hormones, is effective for the treatment of CRS from viral infections, but it can inhibit the immune system, which in turn increases the viral load and aggravates symptoms (Auyeung et al., 2005; Chen et al., 2006). As a novel therapeutic approach, the use of antagonists for specific cytokines such as antagonists for receptors of Anakinra IL-1β and Tocilizumab IL-6 is effective in improving the survival rate of severe sepsis patients (Shakoory et al., 2016; Tanaka et al., 2016). However, these antibodies are limited to a single cytokine species which limits the ability to inhibit inflammation caused by multiple cytokines in CRS.

In general, nanomaterials especially carbon nanomaterials (e.g., carbon black, nanodiamond, graphene, and carbon nanotubes) have a high affinity for binding proteins due to the high surface area to mass ratio and reactive surface properties (Nel et al., 2009). Among carbon nanomaterials, nanodiamond has been proposed as a promising candidate for biomedical applications including drug delivery and imaging because of its unique physicochemical properties such as low-toxic *sp* ^*3*^ diamond structure and fluorescence emission (Lee et al., 2015; Bhattacharya et al., 2016; Gao et al., 2019; Chauhan et al., 2020). Furthermore, in our preliminary studies, the highly-purified detonation-synthesized nanodiamond (DND) showed maximal binding affinity to interleukin-8 (IL-8) inflammatory cytokine *in vitro* (Supplementary Figure S1). Based on this finding, we investigated DND as a cytokine adsorber aiming for rapid suppression of CRS in a sepsis mouse model and for adsorbing inflammatory cytokines in the plasma samples of CRS patients suffering pneumonia, septic shock, and COVID-19.

## Materials and Methods

### Preliminary Study for the Selection of Detonation-Synthesized Nanodiamond Sample as a Cytokine Adsorber

Of seven different DNDs with sequential *sp* ^*3*^ */sp* ^*2*^ carbon ratios in our previous study, the most purified DND sample showed the least cytotoxicity to macrophages and the lowest lung inflammation in rats (Lee et al., 2020a). To evaluate the comparative cytokine adsorption efficacy among seven DND samples synthesized from our previous study (Lee et al., 2020a), we used A549 cells because of their high baseline production of inflammatory cytokine (e.g., 100 pg/ml in an untreated 24 h culture). The cytokine adsorption efficacy test is not for evaluating cytokine production by DNDs but for evaluating the comparative cytokine adsorption efficacy. Each DND sample was treated to A549 cells (The European Collection of Animal Cell Cultures, Porton Down, United Kingdom) at 50, 100, and 200 μg/ml and measured the levels of IL-8 in the supernatant at 24 h after incubation. Carbon black (Printex 90; Evonik Degussa GmbH, Frankfurt, Germany) and Co_3_O_4_ (Nanostructured and Amorphous Materials, Houston, TX, United States) were used as reference particles. The result of this experiment showed that the level of IL-8 was complete depletion by the most purified DND sample only and it was irrespective of serum coating, which suggests that the highly purified DND possesses unique properties for adsorbing inflammatory cytokines without cytotoxicity (Supplementary Figures S1, S2). In this regard, the most purified DND sample among seven DND samples was further evaluated as a cytokine adsorber in CRS. Since the CD and Co_3_O_4_ showed limited capability in the absorption of cytokines *in vitro*, these materials were not tested in the human-sample-based studies.

### Preparation of Detonation-Synthesized Nanodiamond Sample

The highly purified DND samples were prepared according to methods described in our previous study (Lee et al., 2020a). Briefly, 15 g of DND soot (S.W. Chemicals Co., Ltd., Gunsan, South Korea) was mixed with 300 ml of HClO_4_ in a three-neck round-bottom flask connected with a reflux condenser (Graham type) and temperature sensor, and incubated for 24 h at 203°C. It should be noted that the sharp increase of the reaction temperature can produce a violent exothermic reaction at a range of 120–150°C, thus this experiment should be exercised in line with recommendations in the previous report (Lee et al., 2020a). The solution was cooled to room temperature and washed via centrifugation at 7180 *g* for 20 min. The washing steps were repeated five times and then samples were dried overnight in a vacuum oven at 60°C.

### Physicochemical Property Evaluation of Detonation-Synthesized Nanodiamond Sample

The size and shape of the DND sample were evaluated by high-resolution transmission electron microscopy (HR-TEM; Tecnai G2 F20 460 L) following a routine sample preparation process. The *sp* ^*3*^ */sp* ^*2*^ carbon ratio of the sample was evaluated by an inVia UV-Raman microscope (Renishaw, Gloucestershire, United Kingdom) with an excitation frequency of 325 nm (He-Cd laser) in backscattering geometry. To obtain spectra, the integration time and the objective magnification were fixed at 180 s and 100×, respectively. The laser power was maintained within a 10% range. Background subtraction was performed in a range from 900 cm^−1^ to 2000 cm^−1^ by fixed-point subtraction. The specific surface area was measured via nitrogen physisorption using the Brunauer-Emmett-Teller (BET) method (BELSORP-max, BEL Japan, Inc.).

### Dispersion of the Detonation-Synthesized Nanodiamond Sample for *In Vivo* and *In Vitro* Experiments

Due to the hydrophobic nature of the DND, the optimal dispersion protocol was applied with a minor modification to our earlier study (Lee et al., 2020a). Briefly, a stock solution of the DND was prepared by dispersing it in distilled water (DW) at a 10-fold higher concentration of the final working solution and sonicated for 60 min in a bath sonicator (Saehan Sonic, Seoul, South Korea). Then, sterile phosphate-buffered saline (PBS) was added for the working concentration. The zeta potential and hydrodynamic size of the DND sample were evaluated by a Zetasizer-Nano ZS instrument (Malvern Instruments Ltd., Malvern Hills, United Kingdom). Just before administration, the samples were vortex-mixed for 90 s and ultrasonication performed (VC-750; Sonics and Materials, Inc., Newtown, CT, United States) at an amplitude of 20% and pulses of 10 cycles for 20 s on, with 3 s off. The levels of endotoxin contamination were evaluated using a Limulus Amoebocyte Lysate (LAL) assay kit (Cambrex, Walkersville, MD, United States).

### Collection and Preparation of Plasma Samples From Cytokine Release Syndrome Patients

To evaluate the adsorption efficacy of cytokines in plasma samples from CRS patients, blood from pneumonia, septic shock, and COVID-19 patients were collected by the Yeungnam University Medical Center (Daegu, South Korea). Septic shock was defined using criteria provided by the Sepsis Consensus Conference Committee (Singer et al., 2016). Plasma samples from patients diagnosed with the SARS-CoV-2 infection at a Public Health Center (Daegu, South Korea) were collected at the Yeungnam University Medical Center. Ten plasma samples of each disease type were obtained. For negative controls, plasma samples from healthy volunteers were collected (*n* = 10). Clinical data were collected for all patients and reported in our previous study (Lee et al., 2020b). Plasma samples were prepared by centrifugation at 2000 ×*g* for 5 min within 12 h of whole blood collection. The human study protocol was approved by the Institutional Review Board of Yeungnam University Hospital (Daegu, South Korea) (YUH 2018-05-022, 2020-03-057, 2020-05-031-001).

### Measurement of Cytokines in Plasma After Incubation With the Detonation-Synthesized Nanodiamond Sample

For the efficacy validation of DND in the plasma, the stock solution with 10-fold higher concentration of DND dissolved in DW was mixed with human plasma obtained from patients and then reacted at 30 rpm of the rotator to mimic conditions similar to blood flow condition. With the help of agitation, we could perform experiments without precipitation of DND. DND sample was mixed with plasma at 2 μg DND/μg plasma and incubated at 20°C for 15, 30, 45, or 60 min. Then, samples were centrifuged at 23545 ×*g* to pellet DNDs. The concentration of cytokines was measured in the supernatant as well as in DND pellets to detect adsorbed cytokines. The quantification of cytokines was performed using a Human XL Cytokine Array Kit (R&D Systems, Minneapolis, MN, United States) according to instructions provided by the manufacturer. Developed films were scanned, the obtained images were analyzed using ImageJ version 1.43. Levels of inflammatory cytokines including IL-1β, IL-4, IL-6, IFN-γ, and TNF-α were also measured using human Quantikine ELISA kits (R&D Systems, Minneapolis, MN, United States) according to the manufacturer’s protocols.

### Animals and Husbandry

Male C57BL/6 mice (six to seven-week-old, 18–20 g body weight) were obtained from Orient Bio (Seongnam, South Korea) and used after a 12-day acclimatization period. Five animals per cage were housed under controlled temperature at 20–25°C and humidity of 40–45% with a 12:12 h light/dark cycle. Mice were fed a normal rodent pellet diet (5L79, Orient Bio, South Korea) and supplied water *ad libitum*. The mice were maintained and handled according to policies approved by the Institutional Animal Care and Use Committee of the KRIBB (approval no: 20106).

### Cecal Ligation and Puncture Mouse Model

To evaluate the preventive effect of the DND sample in a septic disease setting, the CLP-operated septic mouse model was prepared as previously described (Rittirsch et al., 2009). CLP is a polymicrobial septic model and displays systemic inflammation and excessive production of cytokines in C57BL/6N mice (Seemann et al., 2017). It is worth noting that the CLP model shares the common signaling pathways of sterol regulatory element-binding protein 2 (SREBP2) and nuclear factor κB (NF-κB) with COVID-19, sepsis, and pneumonia for the progression of the CRS (Lee et al., 2020b; Kim et al., 2020; Park et al., 2020). Briefly, a 2-cm midline incision was made to expose the cecum and adjoining intestine. The cecum was then ligated tightly using a 3.0-silk suture 5.0 mm from the cecal tip, punctured with a 22-gauge needle, and then gently squeezed to extrude feces from the perforation site. Then, the intestine was returned to the peritoneal cavity and the laparotomy site was sutured using a 4.0-silk string. For sham operations, all operations except ligation and puncture were carried out. Following the CLP operation, DND samples were administered by intravenous (I.V.) injection at five time-points (3, 6, 9, 12, and 24 h after CLP) at 100 and 200 μg/ml.

### Serum Biochemistry and Histological Analysis of Septic Mice

At 72 h post-CLP, mice were euthanized by collecting blood from abdominal vena cava under deep anesthesia using 3% isoflurane (Forane^®^, Choongwae Pharma. Corp., Seoul, Korea). Then, fresh serum prepared from the whole blood was tested for the serum biochemistry including aspartate aminotransferase (AST), alanine aminotransferase (ALT), blood urea nitrogen (BUN), and creatinine levels using biochemical kits (MyBioSource, San Diego, CA, United States). The levels of inflammatory cytokines including IL-1β, IL-4, IL-6, IFN-γ, and TNF-α were assayed in serum using commercially available mouse ELISA kits according to the manufacturer’s instructions (Thermo Fisher Scientific, MA, United States). Lung samples were fixed with 10% neutral-buffered formaldehyde, and routine histological procedures were performed to evaluate the histological changes.

### WST-1 Cell Proliferation Assay

To assess the cytotoxic effect of the DND, particles were treated to human umbilical vein endothelial cells (HUVECs; Lonza Group Ltd., Basel, Switzerland). In brief, the HUVECs were cultured in endothelial cell growth medium (EGM-2) containing 2% fetal bovine serum (FBS) and supplements (#CC-3162, Lonza Group Ltd., Basel, Switzerland), at 37°C in a humidified 5% CO_2_ atmosphere. The medium was replaced with serum-free EGM-2 medium containing DND (25, 50, 100, 200, or 400 μg/ml) for 24 h to evaluate the dose-response. Then, HUVECs were incubated with DND 200 μg/ml for 12, 24, 36, or 48 h to evaluate the time-response. At the end of indicated time points, WST-1 reagent (10 μL per well; Roche Diagnostics GmbH, Mannheim, Germany) was added to the DND-treated HUVECs, and further incubated for 0.5–4 h at 37°C with 5% CO_2_. Then, absorbance was measured at 480 and 600 nm (background) using a Tecan Spark microplate reader.

### Statistical Analysis

All experiments were independently performed at least three times. GraphPad Prism 7 (GraphPad Software, San Diego, CA, United States) was used for statistical analyses and significant differences were determined by one-way ANOVA. Data are reported as mean ± the standard error of the mean (SEM). *P*-values less than 0.05 were considered statistically significant.

## Results

### Physicochemical Property of the Detonation-Synthesized Nanodiamond Sample

**FIGURE 1:**
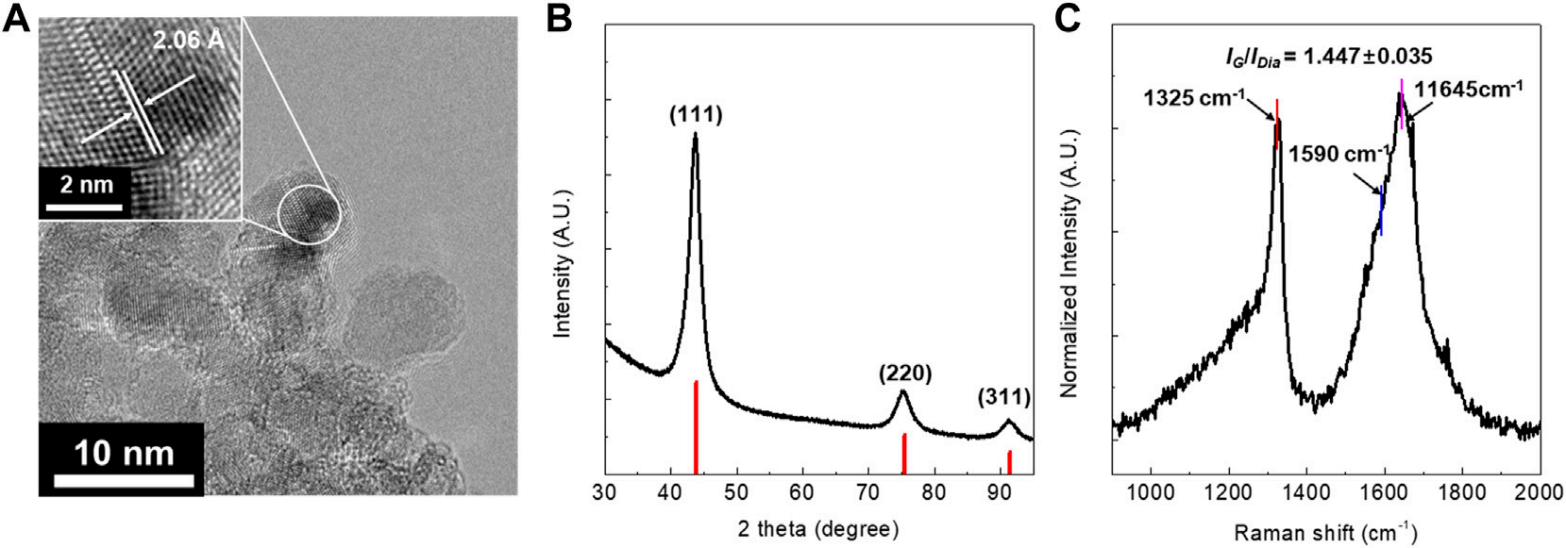
**(A)** HR-TEM image, **(B)** XRD patterns, and **(C)** UV-Raman spectra of the highly purified DND prepared by using HClO_4_. The UV-Raman laser is 325 nm and the intensity ratio was calculated based on *I*_*Dia*_ */I*_*G*_ = 1325 cm^−1^/1590 cm^−1^.

The DND sample was obtained by a harsh chemical purification process using HClO_4_ at 210°C for 24 h (Supplementary Tables S1, S2). As shown in Figure 1A, HR-TEM characterization demonstrates that the size of DND is less than 10 nm does without graphitic shells, which implies that it is highly purified. The lattice fringes with ∼2.06 Å inter-planar spacing correspond to the (111) planes of the diamond (Figure 1A). Furthermore, XRD characterization shows the three most intensive peaks at 43.9°, 75.5°, and 91.5°, which respectively correspond to the (111), (220), and (311) reflections of the diamond (Figure 1B). These results demonstrate that the DND crystals are cubic. The UV-Raman spectrum shows two distinct features: 1) an enhancement of the peak at ∼ 1325 cm^−1^ which is a significant contribution from *sp* ^*3*^ carbon and 2) an upshifted graphite G peak from 1590 cm^−1^ to 1645 cm^−1^ (Figure 1C). Furthermore, the intensity ratio between the diamond peak at 1325 cm^−1^ and the G band at 1590 cm^−1^ (*I*_*Dia*_ */I*_*G*_) was 1.447 ± 0.035 which further supports its high purity (Hong et al., 2018). The surface area measured by BET method was 275.4 m^2^/g.

### The Hydrodynamic Size and Zeta Potential of the Dispersed Detonation-Synthesized Nanodiamond Sample for *In Vivo* Study

The optimal dispersion of nanomaterials is especially important for intravenous injection because agglomerated nanomaterials can block capillaries. The hydrodynamic size of the DND was 173.5 ± 1.8 nm in DW and 280.3 ± 7.0 nm in PBS, which suggests small agglomerates are formed. Although there are various methods in preventing aggregation of nanoparticles such as surface coating (Javed et al., 2020; Shrestha et al., 2020), such methods may block the effective binding sites in the DND particle. Thus, we maintained the pristine state of DND nanoparticles to maximize the protein absorption efficiency. Furthermore, even in the aggregated case, the hydrodynamic size of DND in PBS is still less than 300 nm, there must be no limitations in the systemic circulation. The zeta potentials of the DND sample in DW and PBS were −28.1 ± 0.2 mV and −31.8 ± 0.8 mV, respectively. The DND-therapeutic agent showed no endotoxin contamination.

### The High Binding Affinity of Detonation-Synthesized Nanodiamond With Inflammatory Cytokines in Plasma of Cytokine Release Syndrome Patients

**FIGURE 2:**
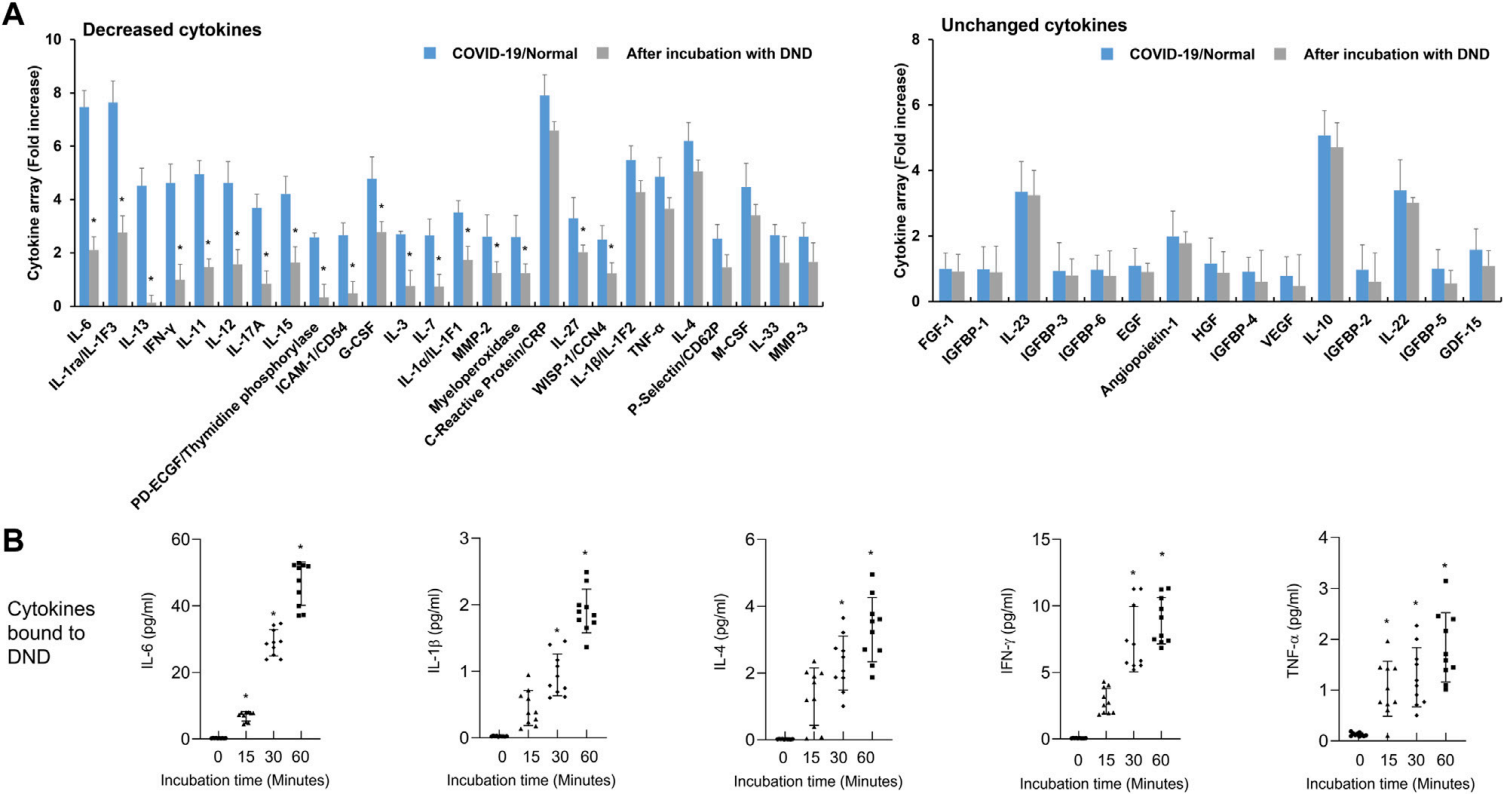
The adsorption efficacy of the highly purified DND with cytokines in plasma samples of COVID-19 patients (*n* = 10). **(A)** Cytokine array showing which inflammatory mediators are adsorbed (left graph) or not (right graph) to the DND from the proteome analysis. **(B)** The adsorption kinetics of DND with key cytokines including IL-6, IL-1β, IL-4, IFN-γ, and TNF-α in the plasma of COVID-19 patients (*n* = 10). Data are mean ± SEM with significance set at ^*^ *p* < 0.05 compared to the control group.

Because the highly purified DND has complete adsorption of high levels of IL-8 normally released by A549 cells without cytotoxic effect (Supplementary Figures S1, S2), we hypothesized that DND could be a useful substance for clearing inflammatory mediators in the blood of CRS conditions such as COVID-19, sepsis, and pneumonia. To evaluate the protective effect of DND for CRS as a cytokine sponge, DND was incubated with plasma from COVID-19 patients for 2 h and the levels of cytokines were analyzed by cytokine array. Various cytokines including interleukins, interferon-gamma (IFN-γ), granulocyte-colony stimulating factor (G-CSF), intercellular adhesion molecule-1 (ICAM-1), matrix metalloproteinases (MMPs), C-reactive protein (CRP), and tumor necrosis factor-alpha (TNF-α) were increased in the plasma of COVID-19 patients compared to the normal group (Supplementary Figure S3). As expected, pro-inflammatory cytokines (CRP, IL-1, IL-4, IL-6, IL-11, IL-12, IL-13, IL-15, IL-17, TNF-α, and IFN-γ), G-CSF, macrophage colony-stimulating factor (M-CSF), MMP, and ICAM-1 were decreased in DND incubated plasma (Figure 2A, left panel). However, IL-10, IL-22, IL-23, angiopoietin, CC chemokine ligand (CCL), chemokine (CXC motif) ligand (CXCL), and growth factors (Figure 2A, right panel) were not altered. Next, as it was speculated that cytokine adsorption was associated with decreased cytokine concentrations in plasma samples, we also measured the kinetics of cytokine binding to DND *in vitro*. The time-course adsorption kinetics of representative inflammatory cytokines are shown in Figure 2B and Supplementary Figure S4. As a result, the levels of IL-6, IL-1β, IL-4, INF-γ, and TNF-α showed time-dependent adsorption kinetics and reached a plateau at 60 min.

**FIGURE 3:**
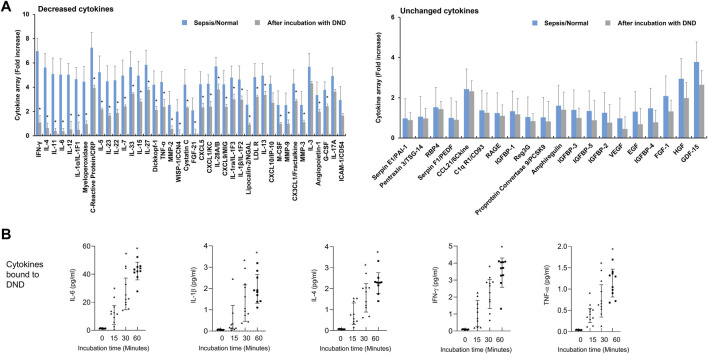
Highly purified DND ameliorates cytokine levels in the plasma of sepsis patients (*n* = 10). **(A)** Cytokine array showing which inflammatory mediators are adsorbed (left graph) or not (right graph) to the DND from the proteome analysis. **(B)** The adsorption kinetics of DND with key cytokines including IL-6, IL-1β, IL-4, IFN-γ, and TNF-α in the plasma of patients with septic shock (*n* = 10). Data are mean ± SEM with significance set at *p < 0.05 compared to the control group.

Next, we evaluated the efficacy of DND for adsorbing cytokines in plasma of patients with septic shock. The inflammatory mediators in plasma of patients with septic shock were significantly increased compared to the healthy control (Figure 3A and Supplementary Figure S5). The incubation of DND with plasma samples of septic shock patients showed a marked reduction of inflammatory mediators such as CRP, IFN-γ, ILs, low-density lipoprotein receptor (LDLR), TNF-α, CXCLs, and MMPs (Gray bar of Figure 3A and Supplementary Figure S5), while CCLs and growth factors did not bound (Right panel of Figure 3A and Supplementary Figure S6). In kinetics analysis, DND started to adsorb in 15 min, and clear time-dependent increased adsorption was observed for IL-6, IL-1β, IL-4, IFN-γ, and TNF-α (Figure 3B). Although DND binds to certain cytokines such as ILs, it is considered that DND may bind to excessive inflammatory mediators in the patients’ blood.

**FIGURE 4:**
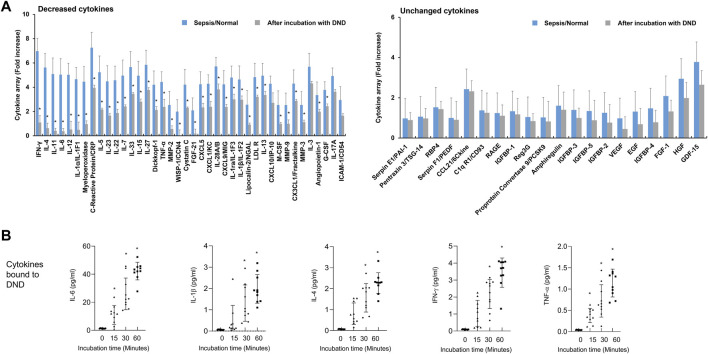
Highly purified DND ameliorates cytokine levels in the plasma of pneumonia patients (*n* = 10). **(A)** Cytokine array showing which inflammatory mediators are adsorbed (left graph) or not (right graph) to the DND from the proteome analysis. **(B)** The adsorption kinetics of DND with key cytokines including IL-6, IL-1β, IL-4, IFN-γ, and TNF-α in the plasma of patients with pneumonia (*n* = 10). Data are mean ± SEM with significance set at *p < 0.05 compared to the control group.

In the next step, we analyzed the adsorption efficacy of DND with the inflammatory mediators in plasma samples of patients with pneumonia. The adsorption pattern in plasma of pneumonia patients was slightly different compared to those of septic shock or COVID-19 (Figure 4A and Supplementary Figure S7). The inflammatory mediators adsorbed to DND in the samples of the pneumonia group were IFN-γ, ILs, MMPs, TNF-α, vascular endothelial growth factor (VEGF), and insulin-like growth factor binding protein-1 (IGFBP1), while DND did not bind to CXCLs, CCLs, and growth factors in the plasma of pneumonia patients (Figure 4A, left panel). As shown from the pneumonia samples (Figure 4B and Supplementary Figure S8), representative proinflammatory cytokines such as IL-6, IL-1β, IL-4, IFN-γ, and TNF-α were adsorbed to the DND irrespective of the disease type. The concentrations of cytokines in plasma samples were reduced approximately 60% within 60 min in a time-dependent manner for all patients’ samples, which suggests DND as a fast-acting pan-cytokine adsorber. Taken together, DND could be used as a therapeutic agent for absorbing inflammatory mediators to ameliorate CRS.

### Preventive Effect of Detonation-Synthesized Nanodiamond for Cytokine Release Syndrome in the CLP Mouse Model

**FIGURE 5:**
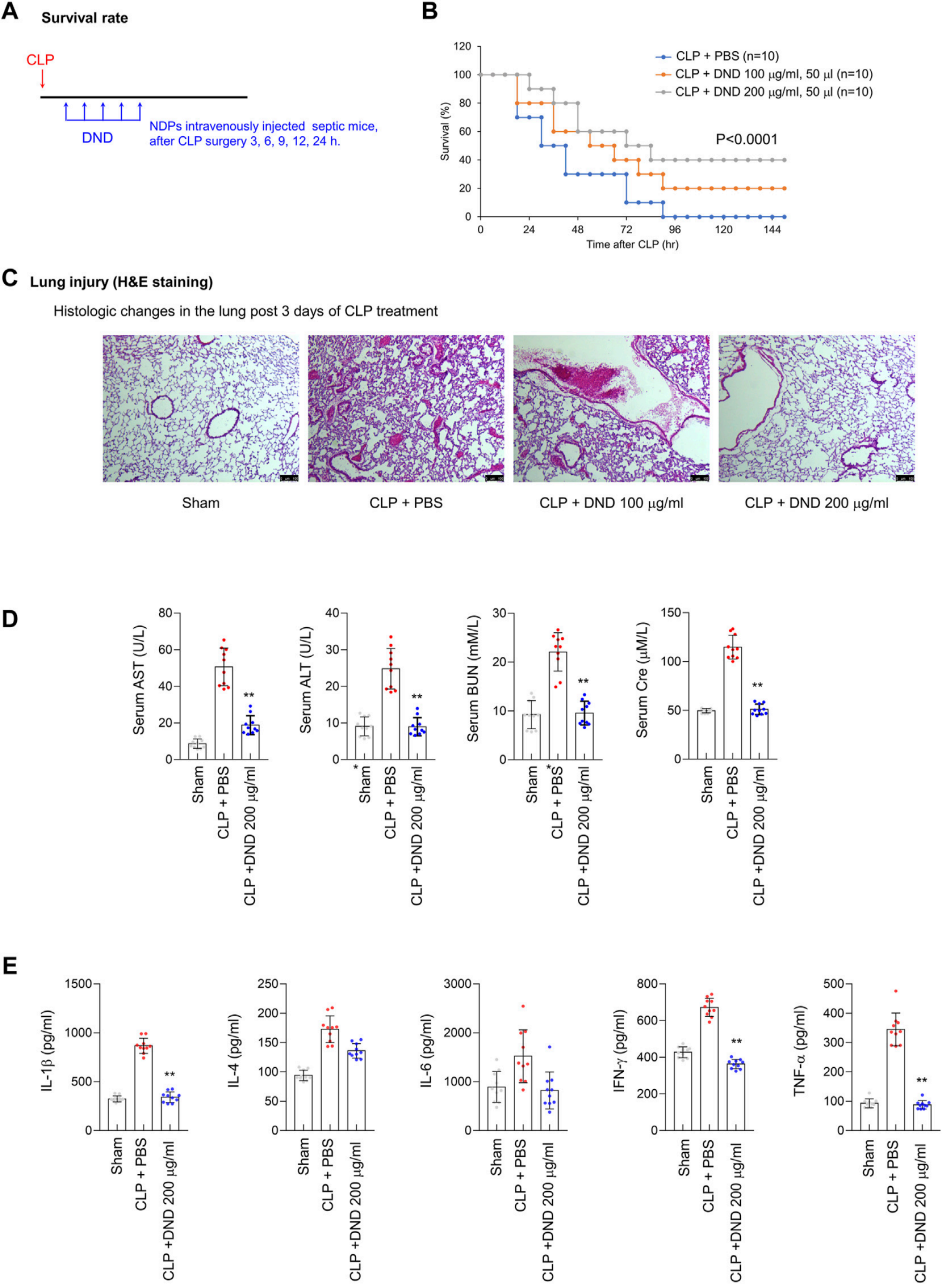
The preventive effect of DND for the CRS condition from a CLP mouse model. **(A)** The timeline of *in vivo* experiments using CLP-operated mice. **(B)** Survival rate of CLP mice after the intravenous administration of DND (*n* = 10 per group). **(C)** Histological analysis of CLP lung tissues after the administration of DND (*n* = 5 per group). **(D)** Serum level of tissue damage markers including aspartate aminotransferase (AST), alanine aminotransferase (ALT), blood urea nitrogen (BUN), and creatinine in mice (**p < 0.01, *n* = 10). **(E)** Serum levels of inflammatory cytokines including IL-1β, IL-4, IL-6, IFN-γ, and TNF-α (*p < 0.05 and **p < 0.01, *n* = 10).

To confirm the preventive effect of DND for CRS conditions, the DND sample was intravenously injected into CLP mice at time intervals of 3, 6, 9, 12, and 24 h after surgery repeatedly (Figure 5A). The survival of CLP mice was improved by 20 and 40% upon administration of 100 and 200 μg/ml of the DND, respectively (Figure 5B). The histological analysis of lung tissue showed that the administration of 200 μg/ml of DND significantly suppressed blood vessel disruption (as noted by reduced blood leakage) and pulmonary inflammation (Figure 5C). Consistent with these observations, serum levels of enzymatic markers of tissue damage including AST, ALT, BUN, and creatine were significantly suppressed after DND administration (Figure 5D). Additionally, the levels of inflammatory cytokines including IL-1β, IL-4, IL-6, IFN-γ, and TNF-α were significantly reduced by DND treatment (Figure 5E). It is noted that the survival rate of CLP mice was increased two-fold in 200 μg/ml of the DND than 100 μg/ml of DND, suggesting its dose-dependent therapeutic effects. However, the interaction between DND nanoparticles and *in vivo* cells needs to be investigated in the future to confirm the safety of DND nanoparticles for the clinical trials.

## Discussion

In this study, we have demonstrated that DND is a potent pan-cytokine adsorber. It is well known that nanoparticles effectively bind proteins to form protein coronae with some specificity exist due to their unique physicochemical properties such as size, surface charge, and hydrophobicity (Breznica et al., 2020). As the size of nanoparticles decreases, the protein binding affinity increases because smaller particles have a higher surface area and a higher surface reactivity compared to their larger counterparts (Cedervall et al., 2007). Thus, the effective protein binding affinity of DND shown in this study could be due to the high surface area of 5 nm-sized DND. The positive surface charge of nanomaterials tends to have a high protein binding affinity, so the surface charge might not be the key to high affinity for the protein-binding as DND used in this study are negatively charged (Gessner et al., 2003; Treuel et al., 2014). However, the electrostatically charging property of the DND can lead to efficient cytokine adsorption (Lee et al., 2011). Lastly, the hydrophobic nature of DND samples may be a key mechanism to explain high affinity protein binding because protein binding affinity increases with the hydrophobicity of nanoparticles (Carrstensen et al., 1992; Kolate et al., 2014; Yu et al., 2020).

The binding between carbon nanomaterials and inflammatory mediators has been reported in previous studies. For example, graphene nanoplatelets showed a promising ability to adsorb inflammatory cytokines such as IL-1β, IL-6, IL-8, and TNF-α (Seredych et al., 2018). Mesoporous carbon materials showed effective removal of IL-8 from human plasma (Pavlenko et al., 2017). Carbide-derived mesoporous carbon displayed adsorption of IL-1β, IL-6, IL-8, and TNF-α from human plasma (Yushin et al., 2006; Yachamaneni et al., 2010). Graphene-based filtration systems also removed cytokines from plasma by adsorbing IL-6, IL-8, and TNF-α (Seredych et al., 2018). Because these studies only tested for the limited cytokines *in vitro* using readily prepared plasma, the possibility for *in vivo* application remains unknown. Here, we demonstrate several favorable features for DND as a CRS suppressor. DNDs began to act immediately after a simple intravenous injection and the experimental data from the CLP mouse model confirmed not only its effectiveness in enhancing rate but also the *in vivo* safety of the DND.

There are possibilities that the DND may bind to cell membrane and regulate cellular metabolism or secretion of cytokines by various mechanisms such as scavenging ROS and changing enzymatic activity (Fresta et al., 2018). In such cases, the cytokine levels will increase the initial state after the irritation of cytokine production by cells unless the DND absorb cytokines. However, in this experiment, the DND indeed reduced the cytokine level in a time-dependent manner, suggesting the absorption of unbound cytokines in plasma. Furthermore, the saturation of binding after 60 min indicates the sufficient biding of cytokines in a given DND mass. This suggests that the highly purified DND could potentially be a novel therapeutic sorbent for treating CRS. To the best of our knowledge, we demonstrate, for the first time, the utilization of carbon nanomaterials for *in vivo* application as a therapeutic agent for CRS.

Until now, diagnostic criteria and standard treatments for CRS are poorly established. Current strategies for CRS treatment include 1) filtration of blood (Frimmel et al., 2014; Greil et al., 2017; Xiao et al., 2019); 2) corticosteroids (Lee et al., 2014; Lee et al., 2019); and 3) antagonists for inflammatory cytokines (Giavridis et al., 2018; Norelli et al., 2018; Sterner et al., 2019). Although these efforts show some benefits in treating CRS, there are several disadvantages such as limited time of action, immune compatibility, and stability (Honore and Matson, 2002; Matson et al., 2004; Schädler et al., 2013; Schadler et al., 2017; van der Poll et al., 2017). Therefore, novel therapeutic approaches are required to treat CRS. In this regard, nanodiamonds are one of the most biocompatible materials because of their inert *sp* ^*3*^ structure, which makes them well suited to biomedical applications (Mochalin et al., 2011). Among nanodiamonds, as the *sp* ^*3*^ */sp* ^*2*^ carbon ratio increases, the less the potential for intrinsic ROS generation was seen (Lee et al., 2020a). Thus, our study demonstrates the feasibility of a highly purified DND as a potent pan-cytokine adsorber, which can prevent CRS. Furthermore, previous studies also showed the safety of nanodiamond as confirmed by the unchanged levels of TNF-α (Tsai et al., 2016) and biocompatibility such as excretion into the urinary tract (Zhu et al., 2012) in the nanodiamond-injected mice. These results collectively indicate the *in vivo* safety of nanodiamond.

Our conceptual idea of removing cytokines using highly purified DND may be employed as a blood filtration agent outside of the body such as for use as a filtration agent in extracorporeal membrane oxygenation (ECMO) (Millar et al., 2016). ECMO was recently granted emergency uses for critically ill patients with COVID-19 from the Food and Drug Administration (FDA) when searched ClinicalTrials.gov (10/07/2021). An additional therapeutic strategy, the adsorption of inflammatory mediators by nanomaterials can be investigated. In this regard, we believe that our study helps to provide evidence for the feasibility of its use as a new cytokine adsorbent in devices such as ECMO, although future studies are necessary to obtain clinical data for this.

## Conclusion

In summary, we have demonstrated that highly purified DND can act as a pan-cytokine sponge in the plasma of CRS patients and the CRS condition of the CLP mouse model. The low-toxicity, fast-acting pan-cytokine absorber, and resulting improved survival rate shown in this study suggest that highly purified DND has great potential for treating CRS *in vivo*, and warrants further studies as a therapeutic application of severe infectious diseases.

## Data Availability

The raw data supporting the conclusion of this article will be made available by the authors, without undue reservation.
