# Supplementary material for: Nanodiamond as a Cytokine Sponge in Infectious Diseases
Source: Front Bioeng Biotechnol. 2022 Apr 4;10:862495. doi: 10.3389/fbioe.2022.862495 (PMC9014093; doi:10.3389/fbioe.2022.862495)
Supplement: Supplementary file 1 [file DataSheet1.docx]

Supplementary Material

**Supplementary Table 1.** The DND sample preparations, including the reaction temperature, processing time, and loading amount.

| Sample name | HClO_4_ | Reaction  temperature | Processing  time | DND  loading amount |
| --- | --- | --- | --- | --- |
| DND 1 | 300 mL | 120 ºC | 24 h | 15 g |
| DND 2 | 300 mL | 135 ºC | 3 h | 15 g |
| DND 3 | 300 mL | 135 ºC | 6 h | 15 g |
| DND 4 | 300 mL | 135 ºC | 15 h | 15 g |
| DND 5 | 300 mL | 135 ºC | 24 h | 15 g |
| DND 6 | 300 mL | 155 ºC | 3 h | 15 g |
| DND 7 | **300 mL** | **210 ºC** | **24 h** | **15 g** |

**Supplementary Table 2.** The concentration (ppm) of metal impurities in the chemically purified DND samples.

| Element | CB | DND 1 | DND 2 | DND 3 | DND 4 | DND 5 | DND 6 | **DND 7** |
| --- | --- | --- | --- | --- | --- | --- | --- | --- |
| Al | 9.08^a^ | 615.20 | 437.30 | 486.60 | 345.40 | 332.50 | 632.60 | **30.45** |
| Co | 0.00 | 0.30 | 0.23 | 0.00 | 0.00 | 0.54 | 0.07 | **0.06** |
| Cr | 0.00 | 2.16 | 5.48 | 1.93 | 0.00 | 0.00 | 0.68 | **3.143** |
| Cu | 2.70 | 5.93 | 13.44 | 8.05 | 3.01 | 0.24 | 1.01 | **0.00** |
| Fe | 19.54 | 157.60 | 366.80 | 217.90 | 158.30 | 97.05 | 143.10 | **1269.00** |
| Mn | 0.81 | 0.22 | 0.73 | 0.26 | 0.25 | 0.00 | 1.15 | **35.60** |
| Ni | 0.63 | 0.00 | 0.09 | 0.00 | 1.31 | 0.00 | 0.00 | **0.00** |
| Pb | 0.00 | 0.00 | 3.98 | 0.00 | 0.00 | 6.62 | 1.97 | **2.98** |
| Zn | 0.00 | 7.16 | 14.67 | 17.14 | 0.00 | 13.91 | 7.14 | **9.99** |
| Total | 32.77 | 788.60 | 842.70 | 731.90 | 508.30 | 450.90 | 787.70 | **1351.00** |

^a^Expressed as ppm (µg/g = metal impurity/DND). CB, carbon black.


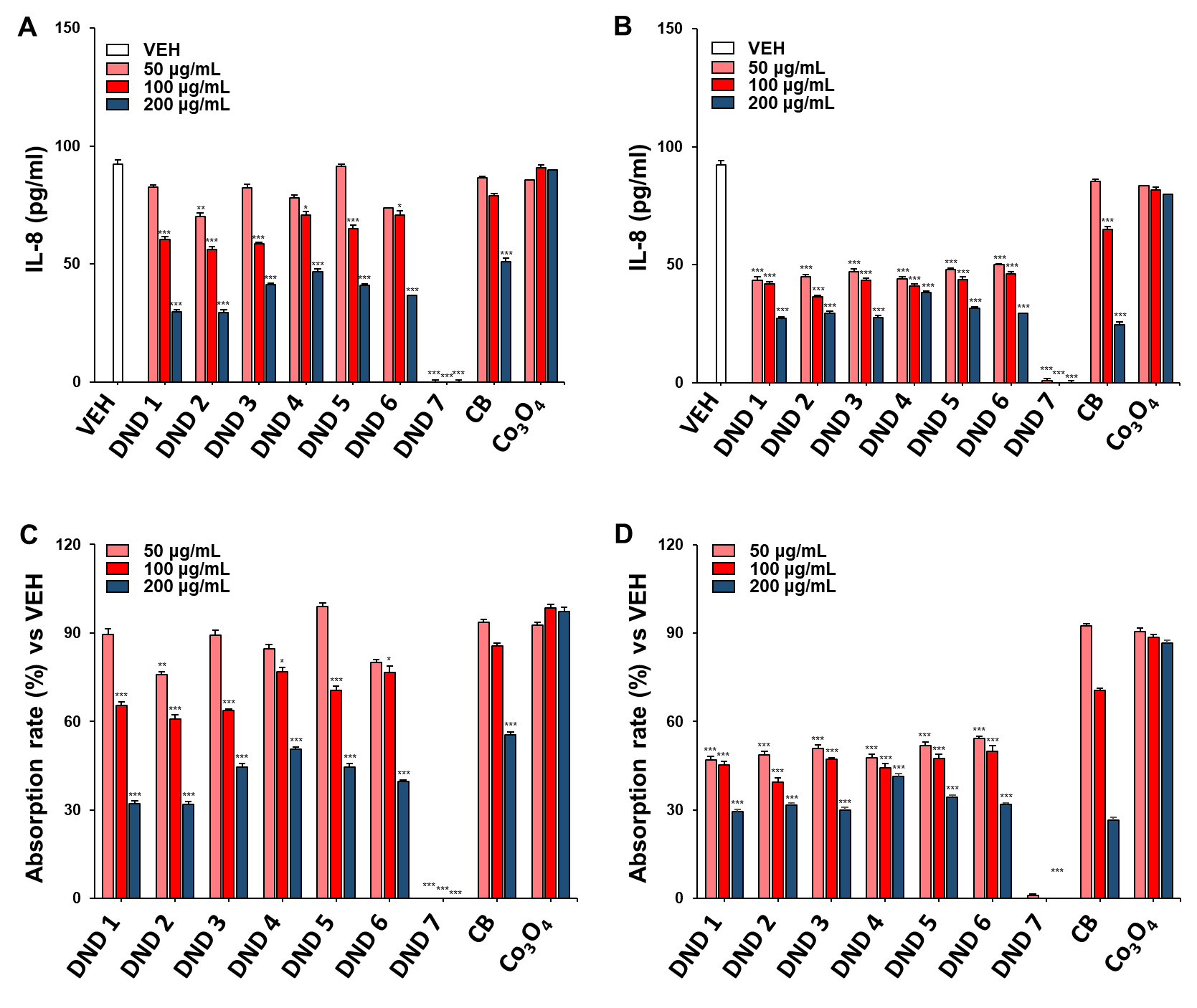


**Supplementary Figure 1** The IL-8 adsorption efficacy of DND samples. At 24 h after treatment of nanomaterials, the levels of IL-8 were measured in cell culture supernatants. (A)The levels of IL-8 after treatment with nanomaterials-pre-coated with fetal bovine serum (FBS). (B) The levels of IL-8 after treatment with manomaterials without FBS pre-coating. Note that the adsorption of IL-8 treated with nanomaterials without FBS coating was much higher than that of nanomaterials with FBS coating. (C) The adsorption rate of nanomaterials pre-coated with FBS versus vehicle control (VEH). (D) The adsorption rate of nanomaterials without FBS pre-coating versus VEH. Note that the DND7, which was named “highly purified DND” in the main experiments, showed complete adsorption of IL-8 regardless of FBS coating, which was contrasted with the other DND samples or reference particles. Values are mean ± SEM, *n*=4 for each group. Significance vs VEH: ^*^p<0.05, ^**^p<0.01, ^***^p<0.001.


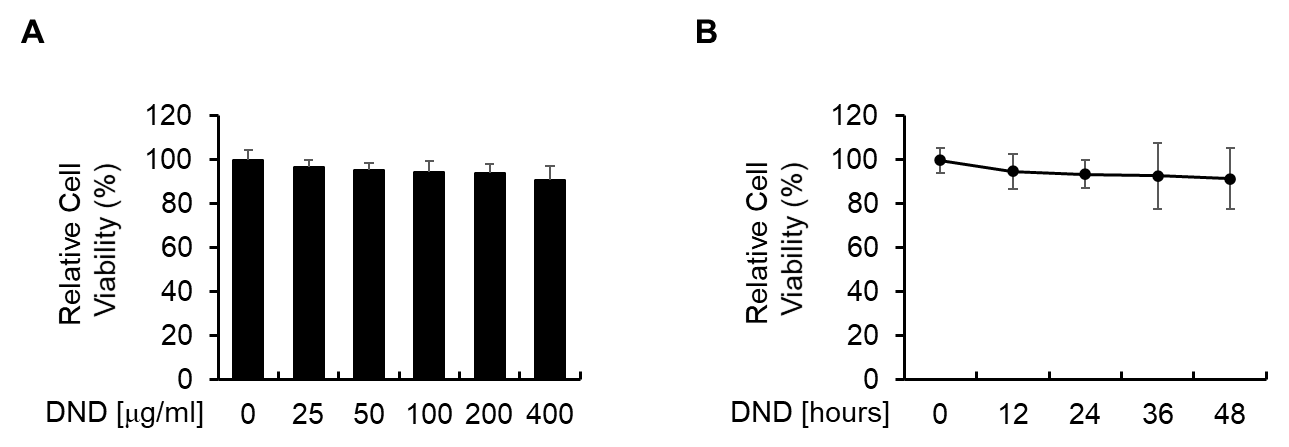


**Supplementary Figure 2.** Cytotoxicity of the highly purified DND was tested dose-dependently (A) or time-dependently (B) in HUVECs.


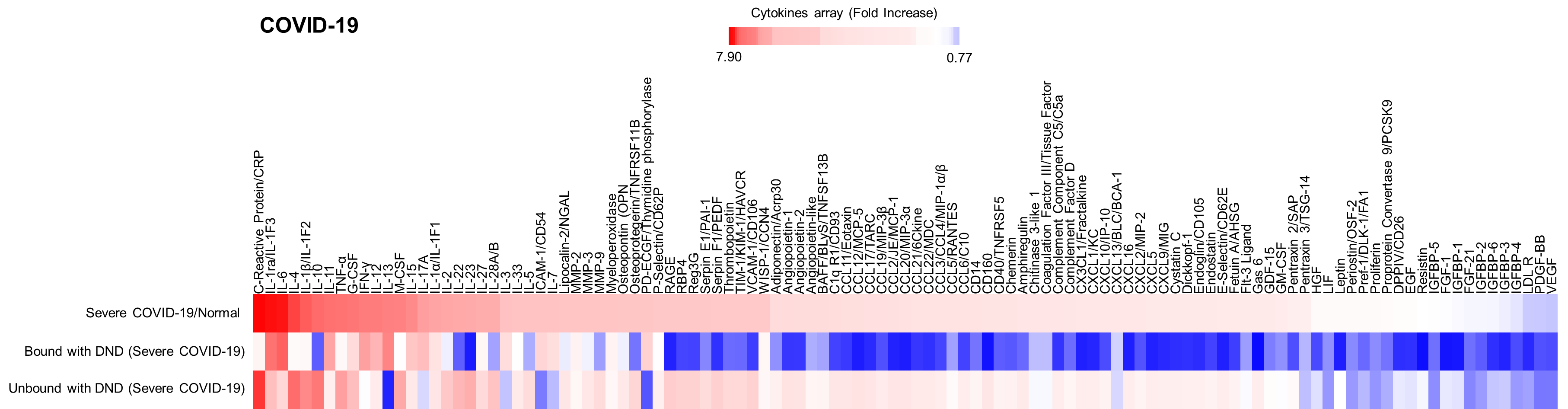


**Supplementary Figure 3.** The levels of inflammatory mediators in plasma of COVID-19 patients (*n*=3) and the levels of inflammatory cytokines after incubation with DND. The levels of inflammatory mediators were measured by the Proteome Profiler Human XL Cytokine Array Kit. The data presented as heatmaps in the top, middle, and bottom panels indicate refer to total cytokine in the plasma, DND-bound cytokines, and DND-unbound cytokines that remain in plasma, respectively.


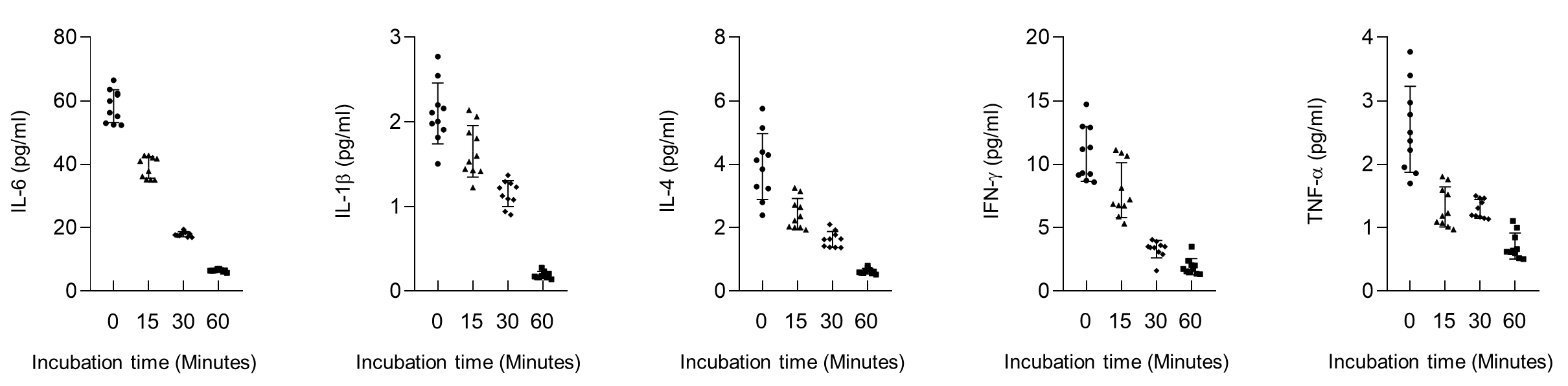


**Supplementary Figure 4.** Incubation time-dependent levels of nanodiamond-unbound cytokines in the plasma of COVID-19 patients (*n*=10). The levels of cytokines in the plasma including IL-6, IL-1β, IL-4, IFN-γ, and TNF-α were analyzed using ELISA kits.


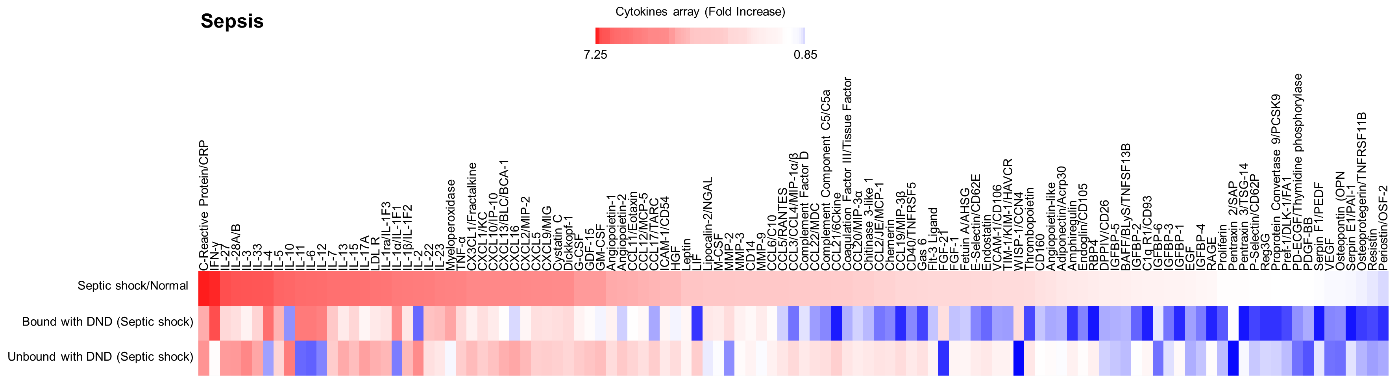


**Supplementary Figure 5.** The levels of inflammatory mediators in plasma of septic shock patients (*n*=3) and the levels of inflammatory cytokines after incubation with DND. The levels of inflammatory mediators were measured by the Proteome Profiler Human XL Cytokine Array Kit. The data presented as heatmaps in the top, middle, and bottom panels indicate refer to total cytokine in the plasma, DND-bound cytokines, and DND-unbound cytokines that remain in plasma, respectively.


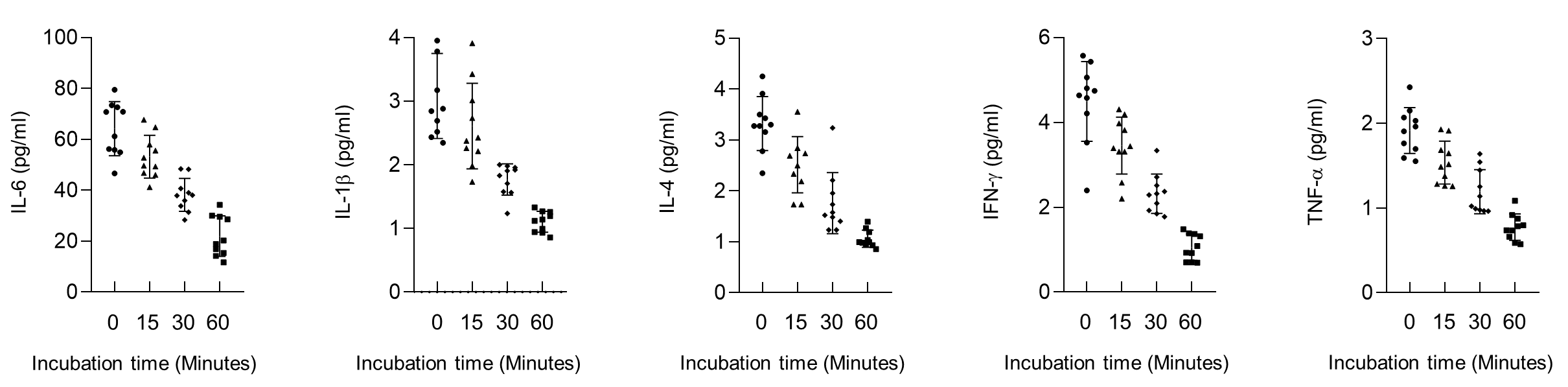


**Supplementary Figure 6.** Incubation time-dependent levels of nanodiamond-unbound cytokines in the plasma of septic shock patients (*n*=10). The levels of cytokines in the plasma including IL-6, IL-1β, IL-4, IFN-γ, and TNF-α were analyzed using ELISA kits.


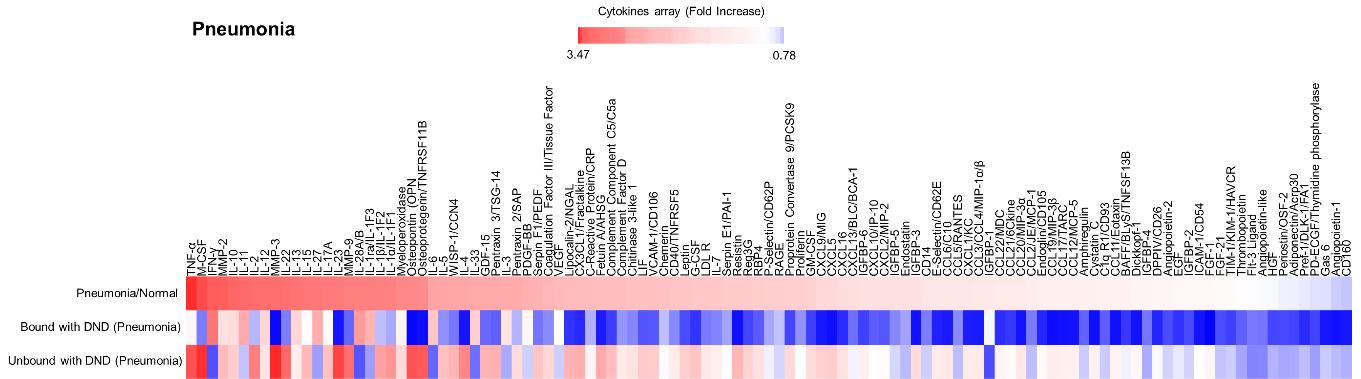


**Supplementary Figure 7.** The levels of inflammatory mediators in plasma of pneumonia patients (*n*=3) and the levels of inflammatory cytokines after incubation with DND. The levels of inflammatory mediators were measured by the Proteome Profiler Human XL Cytokine Array Kit. The data presented as heatmaps in the top, middle, and bottom panels indicate refer to total cytokine in the plasma, DND-bound cytokines, and DND-unbound cytokines that remain in plasma, respectively.


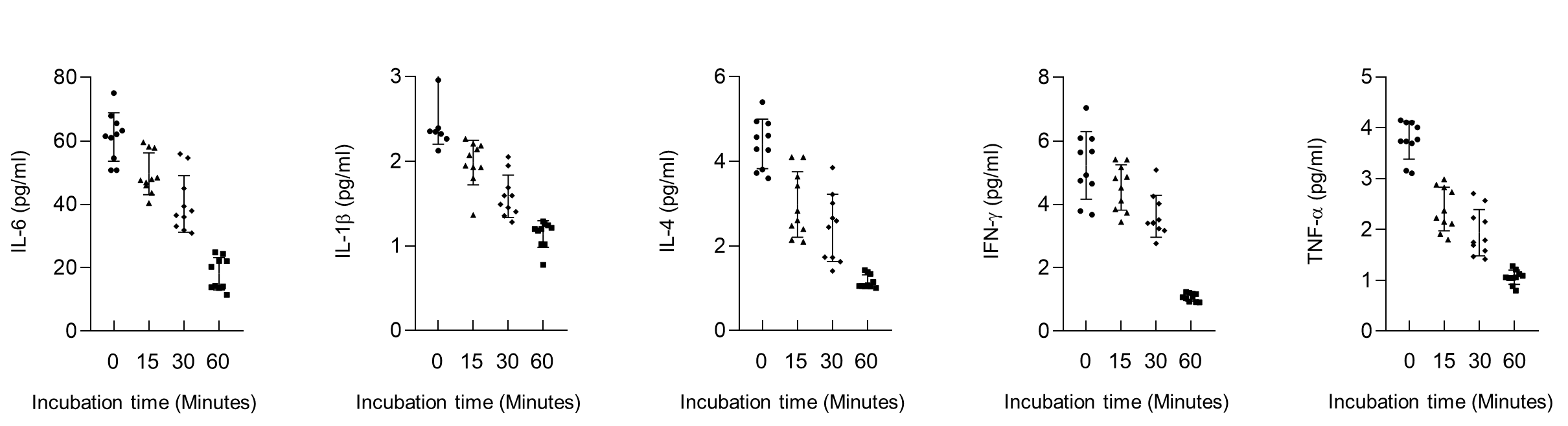


**Supplementary Figure 8.** Incubation time-dependent levels of nanodiamond-unbound cytokines in the plasma of pneumonia patients (*n*=10). The levels of cytokines in the plasma including IL-6, IL-1β, IL-4, IFN-γ, and TNF-α were analyzed using ELISA kits.
